# Detection of Human Adenovirus and Rotavirus in Wastewater in Lusaka, Zambia: Demonstrating the Utility of Environmental Surveillance for the Community

**DOI:** 10.3390/pathogens13060486

**Published:** 2024-06-07

**Authors:** Ngonda Saasa, Ethel M’kandawire, Joseph Ndebe, Mulenga Mwenda, Fred Chimpukutu, Andrew Nalishuwa Mukubesa, Fred Njobvu, Doreen Mainza Shempela, Jay Sikalima, Carol Chiyesu, Bruce Muvwanga, Sarah M. Nampokolwe, Clement Sulwe, Thokozile Khondiwa, Todd Jennings, Ameck Kamanga, Edgar Simulundu, Conceptor Mulube, Wizaso Mwasinga, Jalaimo Mumeka, John Simwanza, Patrick Sakubita, Otridah Kapona, Chilufya Susan Aneta Mulenga, Musole Chipoya, Kunda Musonda, Nathan Kapata, Nyambe Sinyange, Muzala Kapina, Joyce Siwila, Misheck Shawa, Masahiro Kajihara, Ayato Takada, Hirofumi Sawa, Simulyamana A. Choonga, Roma Chilengi, Earnest Muyunda, King S. Nalubamba, Bernard M. Hang’ombe

**Affiliations:** 1 Department of Disease Control, School of Veterinary Medicine, University of Zambia, Lusaka 10101, Zambia; ethelmkandawire@yahoo.com (E.M.); j.ndebe@yahoo.com (J.N.); mukubesaandrew@gmail.com (A.N.M.); millahbruce@gmail.com (B.M.); sarahluyani@gmail.com (S.M.N.); sulweclement4@gmail.com (C.S.); khondiwatk@gmail.com (T.K.); esikabala@yahoo.com (E.S.); wizanso14@gmail.com (W.M.); atakada@czc.hokudai.ac.jp (A.T.); 2 PATH-Zambia, National Malaria Elimination Centre, Chainama Hospital Grounds, Lusaka 10101, Zambia; mchimfwembe@path.org (M.M.); frednjobvu@gmail.com (F.N.); carolchiyesu88@gmail.com (C.C.); tjennings@path.org (T.J.); akamanga@path.org (A.K.); cmulube@path.org (C.M.); emuyunda@path.org (E.M.); 3 Effluents and Pollution Control, Lusaka Water Supply and Sanitation Company, Stand No. 871/2, Katemo Road, Rhodes Park, P.O. Box 50198, Lusaka 10101, Zambia; fredchimpukutu@gmail.com (F.C.); mumejah@gmail.com (J.M.); 4 Churches Health Association of Zambia (CHAZ), CHAZ Complex, Meanwood Drive (off Great East Road), Plot No. 2882/B/5/10, P.O. Box 34511, Lusaka 10101, Zambia; doreen.shempela@chaz.org.zm (D.M.S.); jay.sikalima@chaz.org.zm (J.S.); 5 Macha Research Trust, Choma 10101, Zambia; 6 Zambia National Public Health Institute, Stand 1186, Corner of Chaholi & Addis Ababa Road, Rhodes Park, Lusaka 10101, Zambia; simwanzajohn@gmail.com (J.S.); sakubitap@gmail.com (P.S.); kaponaotridah@gmail.com (O.K.); chilufyam2007@gmail.com (C.S.A.M.); musolechipoya@gmail.com (M.C.); kundagk@yahoo.com (K.M.); nkapata@gmail.com (N.K.); bsinyange@gmail.com (N.S.); mkapina100@gmail.com (M.K.); chilengir@yahoo.com (R.C.); 7 Department of Clinical Studies, School of Veterinary Medicine, The University of Zambia, P.O. Box 32379, Lusaka 10101, Zambia; siwilaj@yahoo.co.uk (J.S.); kshimumbo@yahoo.com (K.S.N.); 8 Hokudai Center for Zoonosis Control in Zambia, School of Veterinary Medicine, University of Zambia, Lusaka 10101, Zambia; misheckshawa@ymail.com (M.S.); kajihara@czc.hokudai.ac.jp (M.K.); h-sawa@czc.hokudai.ac.jp (H.S.); 9 Division of Global Epidemiology, International Institute for Zoonosis Control, Hokkaido University, N20 W10, Sapporo 001-0020, Japan; 10 One Health Research Center, Hokkaido University, N18 W9, Sapporo 001-0020, Japan; 11 Division of International Research Promotion, International Institute for Zoonosis Control, Hokkaido University, N20 W10, Sapporo 001-0020, Japan; 12 Institute for Vaccine Research and Development, Hokkaido University, N21 W11, Sapporo 001-0021, Japan; 13 Ministry of Health, Lusaka Provincial Health Office, 3 Saise Road, P.O. Box 32573, Lusaka 10101, Zambia; simulyamanachoonga@gmail.com; 14 Department of Paraclinical Studies, School of Veterinary Medicine, The University of Zambia, P.O. Box 32379, Lusaka 10101, Zambia; mudenda68@yahoo.com; 15 Africa Centre of Excellence for Infectious Diseases of Humans and Animals, School of Veterinary Medicine, University of Zambia, Lusaka 10101, Zambia

**Keywords:** wastewater, adenovirus, rotavirus, environmental surveillance, concentration methods

## Abstract

Enteric infections due to viral pathogens are a major public health concern. Detecting the risk areas requires a strong surveillance system for pathogenic viruses in sources such as wastewater. Towards building an environmental surveillance system in Zambia, we aimed to identify group A rotavirus (RVA) and human adenovirus (HAdV) in wastewater. Convenient sampling was conducted at four study sites every Tuesday for five consecutive weeks. The research team focused on three different methods of viral concentration to determine the suitability in terms of cost and applicability for a regular surveillance system: the bag-mediated filtration system (BMFS), polyethylene glycol-based (PEG) precipitation, and skimmed milk (SM) flocculation. We screened 20 wastewater samples for HAdV and RVA using quantitative polymerase chain reaction (qPCR) and conventional polymerase chain reaction (cPCR). Of the 20 samples tested using qPCR, 18/20 (90%) tested positive for HAdV and 14/20 (70%) tested positive for RVA. For the genetic sequencing, qPCR positives were subjected to cPCR, of which 12 positives were successfully amplified. The human adenovirus was identified with a nucleotide identity range of 98.48% to 99.53% compared with the reference genome from GenBank. The BMFS and SM flocculation were the most consistent viral concentration methods for HAdV and RVA, respectively. A statistical analysis of the positives showed that viral positivity differed by site (*p* < 0.001). SM and PEG may be the most appropriate options in resource-limited settings such as Zambia due to the lower costs associated with these concentration methods. The demonstration of HAdV and RVA detection in wastewater suggests the presence of the pathogens in the communities under study and the need to establish a routine wastewater surveillance system for the identification of pathogens.

## 1. Introduction

Enteric viruses pose a significant global public health concern due to their association with acute gastroenteritis [1]. These include human adenoviruses (HAdV), Noroviruses, group A rotavirus (RVA), Hepatitis A virus (HAV), and Enteroviruses [2]. Therefore, it is imperative to investigate the potential environmental sources of these infections to develop efficient intervention strategies. Given the increasing scarcity of water worldwide, there is the risk of insufficiently treated wastewater ending up in agriculture fields or in residential homes, which can pose a significant health risk [3]. The ability to detect pathogens is essential because of the occasional deficiencies in wastewater treatment techniques against viruses [4]. Therefore, wastewater surveillance is central in identifying the potential risk areas associated with wastewater.

Markers of potential enteric viral pathogen presence are required for any intervention to yield meaningful surveillance results [5]. Since HAdV is widely present and stable in the environment, the virus is currently considered a reliable indicator [1,5]. The adenovirus is a good candidate as an indicator due to its abundance and ease of detection. HAdV is also associated with a wide range of illnesses affecting the respiratory [6] and gastrointestinal [7] systems of both symptomatic and asymptomatic subjects. These features of the adenovirus result in a high concentration of the virus being excreted in the feces. Besides being a viral indicator, HAdV remains one of the main causes of diarrhea in both immunocompromised adults and younger populations [8]. The adenovirus is a double-stranded, non-enveloped DNA virus belonging to the family *Adenoviridae* and comprises six genera (*Mastadenovirus*, *Aviadenovirus*, *Atadenovirus*, *Siadenovirus*, *Ichtadenovirus*, and *Testadenovirus*) [3]. The mastadenoviruses are responsible for most human infections. Currently, seven species have been identified as human pathogens, namely human mastadenovirus A-G [3]. The human mastadenoviruses A, B, and C are implicated in respiratory infections, while the human mastadenovirus F serotypes 40 and 41 cause acute gastroenteritis in children less than 1 year old [1]. To date, more than 110 serotypes have been identified [1,3].

The rotavirus is also a common cause of acute gastroenteritis, typically introduced into the wastewater by infected individuals [9]. The virus belongs to the genus *Rotavirus* in the family *Sedoreoviridae* in the order Reovirales. The genus *Rotavirus* is classified into distinct species, rotavirus A-J, with RVA the most common cause of human infections. While vaccines for RVA have been developed and adequately administered in Zambia [10], the reports highlight low efficacy, particularly in developing countries [11,12]. Zambia introduced the Rotarix™ vaccine in 2012, and this G1P [7] monovalent, live-attenuated vaccine has demonstrated adequate protection against severe disease [13,14].

The presence of HAdV and RVA viral pathogens in wastewater presents the opportunity to establish a surveillance system that can track viral pathogens and estimate their prevalence, genetic diversity, and geographic distribution [14,15]. Wastewater surveillance can help to detect viruses that are not evident using clinical surveillance [16]. The surveillance of viruses in wastewater also provides an opportunity to detect the spread of infections in areas where the resources for clinical diagnosis and reporting systems are limited or unavailable [17]. It is also valuable for identifying newly introduced viruses in populations, and for tracking the fluctuations and outbreaks resulting from seasonal or precipitation changes [14,18]. For this purpose, wastewater surveillance can form an integral part of the “early warning” system [19,20].

In Zambia, diarrhea-associated deaths are ranked third among children under five [21]. Notably, HAdV and RVA are among the top six enteric pathogens associated with moderate-to-severe diarrhea in Zambia, although the magnitude of HAdV infection remains unclear [22]. Thus, wastewater surveillance can aid in monitoring the effectiveness of the current interventions in mitigating the prevalence of enteric pathogens. To our knowledge, no report on the detection of HAdV and RVA in wastewater has been previously published in Zambia. This study set out to detect HAdV and RVA in wastewater and provide a proof-of-principle for the establishment of a robust and cost-effective surveillance system for viral pathogens of public health importance in Zambia.

## 2. Materials and Methods

### 2.1. Study Design

This prospective cross-sectional study was conducted in Lusaka in a wastewater network system to detect the presence of HAdV and RVA. The study was conducted at four sewerage sites that service selected parts of the Lusaka District (Table 1). The sites included two pump stations (Chelstone and Mass Media), one stabilization pond (Kaunda Square), and one wastewater treatment plant (Manchichi).

### 2.2. Sample Collection

Convenient sampling was employed for this study. The collection at the four sites was carried out between 7 am and 9 am by four sampling teams for five consecutive Tuesdays from 25 July 2023 to 22 August 2023. At each site, a grab-composite sample (6 L) was constituted over a period of 1 h. The composite sample was aliquoted into separate bottles for the three concentration methods: (i) the bag-mediated filtration system (BMFS), (ii) skimmed milk (SM) flocculation, and (iii) polyethylene glycol precipitation. The sample bottles were placed in ice-chilled cool boxes and transported to the University of Zambia, School of Veterinary Medicine, Department of Disease Control BSL2 laboratory for processing. Upon arrival at the laboratory, the samples were immediately prepared for concentration.

### 2.3. Viral Concentration

The wastewater samples were subjected to three viral concentration methods before the nucleic acid extraction.

#### 2.3.1. Skimmed Milk Flocculation

A composite wastewater sample (500 mL) was used for the direct skimmed milk flocculation method. Briefly, the skimmed milk powder (0.5 g) was dissolved in 50 mL of sterile water to obtain a 1% (*w*/*v*) skimmed milk solution [23]. The pH of the solution was carefully adjusted to 3.5 using 1 M hydrochloric acid (HCl). Five mL of the 1% skimmed milk solution was added to 500 mL of the wastewater composite sample to obtain a final concentration of 0.01% (*w*/*v*). The samples were stirred for 8 h, and the flocs were allowed to settle at room temperature for another 8 h. The supernatant was carefully removed with the serological pipettes without agitating the flocs. A final volume of 50 mL containing the flocs was transferred to the 50 mL falcon tubes and centrifuged at 8000× *g* for 30 min. The supernatant was carefully removed, and the pellets were carefully dislodged. The pellets were resuspended in 2 mL of 0.2 M phosphate buffer saline (PBS) at pH 7.5. The final concentrated sample was aliquoted in small working volumes (~200 μL) and stored at −80 °C until the viral nucleic acid extraction.

#### 2.3.2. Bag-Mediated Filtration System (BMFS)

The samples were collected into the BMFS bag and processed as described previously [24]. Briefly, the wastewater (6000 mL) from the composite sample was collected in the sampling bags with a pre-screen mesh (249-μm pore size) over the opening. The composite sample was filtered into the collection bag mounted on a custom-made tripod stand (BoundaryTEC, Minneapolis, MN, USA). A ViroCap^TM^ filter (Scientific Methods, Inc., Granger, IN, USA) was attached to the bag’s outlet port and the sample was allowed to flow through the filter by gravity. The average volume filtered was about 5400 mL, over an average of 90 min to 3 h (the time for the filtration was dependent on the amount and size of the fecal debris in the wastewater). The filter was transported under a cold chain to the University of Zambia for viral elution and secondary concentration with skimmed milk. To elute the virus from the viral cup filter, the beef extract [1.5% of beef extract and 0.05 M glycine solution (Tokyo Chemical Industries Co, Tokyo, Japan) at pH 9.5] was injected into the filter inlet and incubated for 30 min before recovering the eluate through the filter outlet using a peristaltic pump.

##### Secondary Concentration Procedure

The skimmed milk secondary flocculation procedure was conducted at the laboratory. A total of 3 mL of 1% *w*/*v* skimmed milk was added to the eluate. The pH of the mixture was adjusted to the range of 3.0–4.0 using sodium hydroxide (NaOH) and HCl. To coagulate the proteins in flocs, the eluate was incubated at room temperature and shaken evenly for 2 h. The sample was aliquoted into 50 mL falcon tubes and centrifuged at 3500× *g*, 4 °C for 30 min. The sample was returned to the biosafety cabinet (BSC) and the supernatant was decanted. The pellets were completely resuspended in 10 mL of sterile PBS at pH 7.4 using vigorous vortexing. The concentrated sample was aliquoted in 1 mL working volumes and stored at −80 °C until use.

#### 2.3.3. Polyethylene Glycol-Based (PEG) Concentration

PEG precipitation was performed as previously described [25]. Briefly, 14 g of PEG 8000 and 1.17 g of NaCl were added to 100 mL of the composite sample. The PEG 8000 and NaCl were completely dissolved using vigorous shaking. The sample was stirred at 200 rpm, 4 °C for 4 h followed by centrifugation at 6500× *g*, at 4 °C for 30 min. The supernatant was discarded, and the pellet was re-suspended in 6 mL of sterile PBS (pH 7.4). The final concentrated sample was aliquoted in small working volumes (~200 μL) and stored at −80 °C until use.

### 2.4. Nucleic Acid Extraction

Approximately 200 µL of the sample was extracted using Qiagen QIAamp^®^ mini-RNA or DNA kits (QIAGEN, Germantown, MD, USA). The total nucleic acids were eluted in 60 µL of the elution buffer according to the manufacturer’s instructions. To minimize freezing and thawing, each sample was aliquoted into 20 µL volumes and stored at −80 °C until use.

### 2.5. Genomic Screening for Adenovirus and Rotavirus

#### 2.5.1. Detection of Adenovirus and Rotavirus on PCR

HAdV and RVA were detected using a one-step TaqPath qPCR protocol following the manufacturer’s instructions. Briefly, 20 µL total volume of each reaction was prepared to consist of the following: 1 µL of the primer and probe mix (Table 2), 15 µL of the TaqPath Master Mix, 2 µL of the total nucleic acid sample, and 2 µL of nuclease-free water. The mixture was run under the following conditions: pre-incubation at 25 °C for 2 min, followed by reverse transcription incubation at 50 °C for 15 min, enzyme activation at 95 °C for 2 min, 40 cycles of amplification with a two-step annealing and elongation at 95 °C for 3 s, and 60 °C for 30 s. For the HAdV detection, the reverse transcription incubation step (of 50 °C for 5 min) was omitted in the run protocol, since HAdV is a DNA virus. Nuclease-free water was used as a negative control and a sample was considered positive for HAdV and RVA when the cycle threshold (Ct) value was less than 32.

#### 2.5.2. Conventional PCR and Sequencing of Adenovirus

To detect the presence of HAdV in wastewater, two PCR reactions targeting the hexon gene were performed using the Quick Taq Hs Dye Mix (TOYOBO, Osaka, Japan), following the manufacturer’s instructions using the primers in Table 2. The initial reactions did not include any positive control, as it was not available. The initial PCR targeting the hexon gene was performed with the primer pairs Hex1DegFw and Hex2DegRv, yielding 301 bp product. The amplicon was identified as a HAdV sequence, which was subsequently used as a template for designing the primers for the next PCR. The second PCR overlapping the capsid and hexon genes was performed using the primer pairs, Adeno Cap17467Fw and Adeno Hex 18185Rv, yielding a 718bp amplicon. Both PCRs were performed with the conditions: 94 °C for 2 min, 94 °C for 30 s, 54 °C for 30 s, 68 °C for 1 min, and final extension at 68 °C for 5 min. Nuclease-free water was used as a negative control for all the cPCR reactions. The PCR products for the first and second PCRs were observed on a 1% agarose gel stained with ethidium bromide.

The PCR products were purified using the Promega PCR Product and Gel Purification Kit (Promega, Madison, WI, USA). Subsequently, the purified PCR products were sequenced using the Big Dye Cycle Sequencing kit V3.1. The successful sequences were processed on a 3500 Genetic Analyzer (Applied Biosystems, Foster City, CA, USA). The nucleotide sequences were analyzed using Genetyx Version 12.0 (GENETYX Corporation, Tokyo, Japan) and deposited in the National Center for Biotechnology Information (NCBI).

### 2.6. Statistical Analysis

Data were summarized using the R package dplyr v1.0.7 (RStudio, Boston, MA, USA) and visualized using the basic R program. Additionally, a comparison of the positivity between sites was carried out using ANOVA to test for overall significance (*p* ≤ 0.05). To determine which site pairs were different, Tukey’s HSD test was applied for multiple comparisons (*p* ≤ 0.05). The percentage identity was obtained by comparing the HAdV Hexon gene with the reference sequences from GenBank.

## 3. Results

### 3.1. Detection of Adenovirus and Rotavirus

Of the 20 samples tested on qPCR, 18/20 (90%) tested positive for HAdV and 14/20 (70%) tested positive for RVA (Table 3). The 18 positives for HAdV were distributed by site as follows: Chelstone (*n* = 3), Kaunda Square (*n* = 5), Mass media (*n* = 5), and Manchichi (*n* = 5) (Table 3). Equally, for RVA, the 14 positives consisted of Chelstone (*n* = 3), Kaunda Square (*n* = 4), Mass Media (*n* = 4), and Manchichi (*n* = 3). On conventional PCR for HAdV, 16/20 were positive, consisting of samples from Chelstone (*n* = 3), Kaunda Square (*n* = 5), Mass Media (*n* = 3), and Manchichi (*n* = 5) (Table 3).

**Figure 1:**
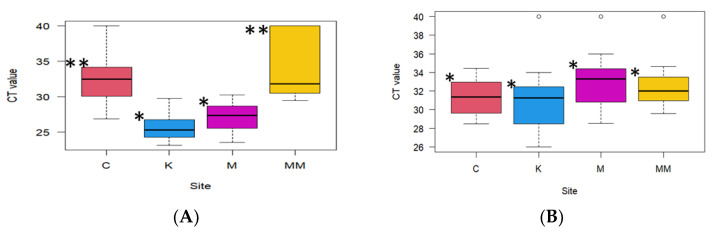
HAdV (**A**) and RVA (**B**); Analysis of the difference in the positivity on qPCR on the four study sites, namely: Chelstone (C), Kaunda Square (K), Manchichi (M), and Mass Media (MM). No significant difference between similar asterisks; (* or **). Significant difference between sites with different asterisks.

The difference in HAdV detection between the four study sites was evaluated for significance by pairwise comparison of the sites. Kaunda Square and Chelstone, Manchichi and Chelstone, Kaunda Square and Mass Media, and Manchichi and Mass Media showed significant differences in the detection of HAdV (Figure 1A). RVA did not show a significant difference in positivity in all the sites (Figure 1B).

Of the conventional PCR-positive HAdV samples, 11/18 were successfully sequenced and BLASTn revealed a nucleotide sequence similarity to HAdV, with the percentage identity ranging from 98.48% to 99.53% (Table 4). For the gene sequencing, a conventional two-step RT-PCR was performed and none of the RVA qPCR samples were successfully amplified.

### 3.2. Comparison of Viral Concentration Methods

**Figure 2:**
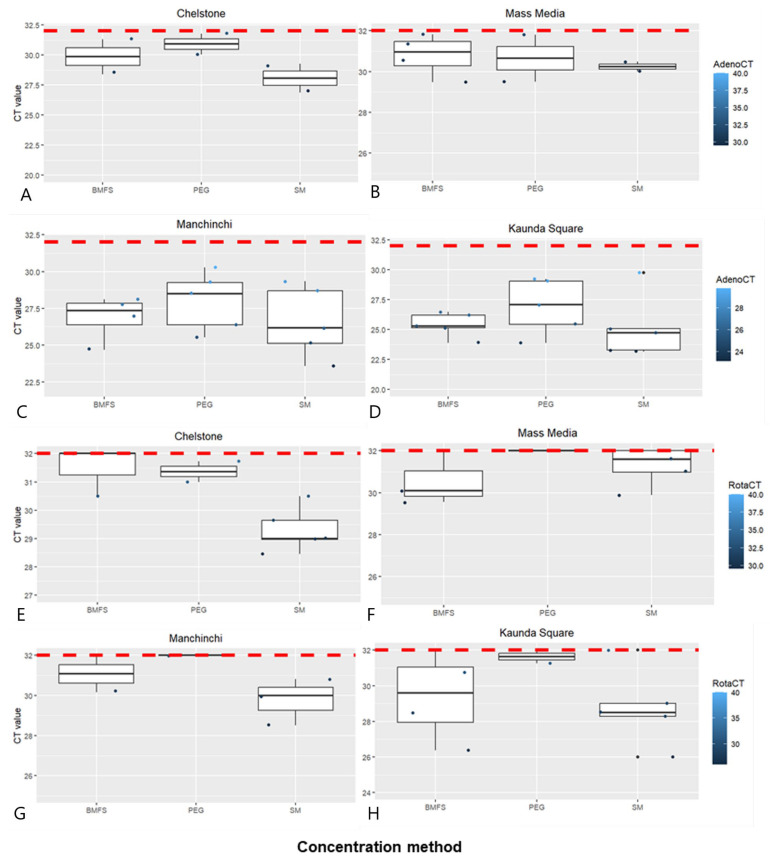
Comparison of viral concentration methods for the detection of HAdV (**A**–**D**) and RVA (**E**–**H**) using qPCR in wastewater at the four sites of collection, based on the cycle threshold of 32.

We examined the effectiveness of three viral concentration methods on HAdV and RVA: BMFS, PEG precipitation, and SM flocculation. The results from the three methods (BMFS, PEG, and SM) showed that the effectiveness varied from site to site (Figure 2). SM flocculation showed consistently lower Ct values for HAdV (Figure 2A–D) and RVA (Figure 2E–H) for all four sites. The BMFS Ct values for Mass Media/HAdV were higher than for SM (Figure 2). Likewise, PEG showed the highest Ct values for both HAdV and RVA. Of the 20 samples, BMFS and SM flocculation had 14/20 (70%) HAdV positives each, while PEG precipitation had 13/20 (65%) (Supplementary Table S1). On the other hand, SM flocculation (10/20, 50%) was most consistent on RVA positivity from all the study sites, followed by PEG (6/20, 30%) and BMFS (7/20, 35%) (Figure 2 and Supplementary Table S1). However, the difference between the concentration methods was not statistically significant.

## 4. Discussion

We investigated the presence of HAdV and RVA in wastewater as a novel method for the surveillance of enteric pathogens in Zambia; to our knowledge, this is the first report of RVA and HAdV detection from wastewater in the country.

Our objective of detecting these viruses was met by screening 20 influent wastewater samples from four sites over the course of five weeks; 18 samples were positive for HAdV, a prevalence of 90%, and 14 samples were positive for RVA, a prevalence of 70%. The results were consistent with the global trend, which showed that HAdV is more prevalent in wastewater than RVA [5]. Notably, the prevalence of HAdV was higher than that obtained in other African countries, including South Africa (64%), Morocco (45.5%), and Egypt (67%) [3,9]. However, when compared with other regions outside Africa, such as Brazil (100%), Norway (92%), the US state of Michigan (99%), and Greece (92.3%), the prevalence of HAdV was lower [1,3]. This variance could be attributable to differences in the intervention strategies, sampling, and detection methods [27,28].

The RVA prevalence of 70% was close to the global prevalence of RVA of 38–67% [29], hence this is a significant finding because diarrhea-associated death is among the major health problems globally. Recent reports have shown that one in every 260 children born dies from RVA-associated infections [18,29]. The detection of RVA, a regularly implicated acute gastroenteritis-causing pathogen in children is critical in developing effective control measures in communities. Using wastewater surveillance to identify high-risk areas, the negative public health impact may be contained and cleared; however, several attempts to amplify the rotavirus genome for genetic sequences could have been unsuccessful, probably due to the low quality of RNA.

Zambia’s implementation of national water reforms initiated in the 1990s saw the creation of commercial utilities (CUs) in urban and peri-urban areas to provide sanitation services. The vast majority of the population depends on pit latrines. CUs collect sewage using a network of sewers from serviced areas and vacuum tankers from unserviced areas and treat it using conventional (mechanized sewage treatment plants) and unconventional treatment methods (sewage stabilization ponds). Sewage treatment facilities treat sewage to the required Zambia Environmental Management Agency (ZEMA) standards before it is discharged into the aquatic environment. For the purpose of the study, influent wastewater samples were collected prior to treatment.

The high level of detection of these two viruses in wastewater suggests that the surveillance of wastewater can help to identify areas of potential disease outbreak before they manifest in health facilities. Wastewater surveillance can therefore provide public health officials with an early warning signal and a better understanding of the prevalence of the pathogens in communities. This is important because HAdV and RVA are among the most implicated enteric viruses in children, with HAdV responsible for respiratory infection and RVA for acute gastroenteritis [3,29].

We compared three viral concentration methods for wastewater analysis. All the methods were effective—there was no statistically significant difference for both viruses, although PEG was relatively the least effective in comparison. Some studies have suggested that PEG precipitation was an effective viral concentration method [2,30,31]. Other viral concentration methods, such as ultracentrifugation, have been reported as effective in the wastewater viral concentration, although they were not included in the study due to unavailability and the high cost involved [27]. Because of the lower costs associated with SM and PEG, these methods may be recommended for routine surveillance in resource-limited settings, despite the latter being less effective compared with BMFS.

We also compared the number of positives per site. The viral shedding variance by site suggested the benefit of targeting disease prevention and mitigation interventions in the areas serviced by the Manchichi and Kaunda Square treatment sites, communities with a potentially greater risk of enteric pathogens [14,32]. This is critical in guiding policy towards the control of pathogenic enteric viruses and other related public health illnesses in Zambia and in other areas with limited resources.

This study was limited by the small sample size and the short duration of sampling, which did not allow for an in-depth analysis of the detection and yield of the concentration methods employed in the study. The detection of the viral genome in wastewater is an indication of the circulation of the viruses in the population served by the sewer system. It will be important to carry out further tests over a longer period in the communities to verify the variations in the genome quantities observed. Further testing of these and other available concentration methods will need to be carried out to establish the sensitivity prior to adoption.

## 5. Conclusions

The detection of HAdV and RVA in Zambian wastewater has been reported for the first time in this investigation. The presence of the two viruses is an indication of the abundance of the viruses in the community that is serviced by these watersheds. Wastewater surveillance provides an opportunity for the remote monitoring of organisms of public health significance and an early warning signal of potential disease outbreaks. This study tested three concentration methods and found that all were effective in detecting HAdV and RVA. Due to the relatively low cost, SM flocculation and PEG precipitation are options for routine surveillance in settings such as Zambia. Overall, environmental surveillance can be a cost-effective system to track wastewater-borne pathogens and identify that communities at risk of enteric virus outbreaks.

## Figures and Tables

**Table 1:** The sewer catchment areas serviced by each of the four wastewater treatment facilities in the Lusaka District.

Site	Sewer Catchment Area
Chelstone pumping station	Chelstone Zambia Airways, Waterfalls mall
Kaunda Square stabilization ponds	PHI, Nyumba Yanga, Ibex Hills, Chamba Valley, Chainama, Kaunda Square Stage 1, Kaunda Square stage 2, Mtendere East, Salama Park, Mtendere, part of Kabulonga, part of Woodlands, Helen Kaunda, part of Kalingalinga, East Park mall, part of Handsworth
Mass Media pumping station	University of Zambia, part of Showgrounds, part of Longacres, Mass Media, Arcades mall up to National Assembly
Manchichi sewage treatment plant and Garden stabilization ponds	Part of Woodlands, Chilenje, Libala, Villa Elizabetha, Kabwata, Northmead, Parts of Emmasdale, part of Rhodes Park, Mass Media, part of Central Business district, Thorn Park, Part of the Light Industrial Area bordered by Great North Road, Sheki Road, Lumumba Road, and Kalambo Road.

**Table 2:** Primers used for qPCR (1–3; 8–10) and cPCR (4–7) amplification of HAdV (1–7) and RVA (8–10).

No.	Name	Sequence	Tm (°C)	Reference
1	JTVXF_18895Fw	5′-GGACGCCTCGGAGTACCTGAG-3′		[26]
2	JTVXR_18990Rv	5′-ACIGTGGGGGTTTCTGAACTTGTT-3′	60	
3	Probe JTVXP_18923Fw	5′-CTGGTGCAGTTCGCCCGTGCCA-3′		
4	Hex1DegFw(1)	5′-GCCSCARTGGKCWTACATGCACATC-3′	54	[23]
5	Hex2DegRv(1)	5′-CAGCACSCCICGRATGTCAAA-3′		
6	Adeno Cap 17467Fw(2)	5′-CTGTAGGTTCCGTTCCCGTT-3′	54	
7	Adeno Hex 18185Rv (2)	5′-CGGTGCCTGAGCAAAGGTAT-3′		
8	RotaA-NSP3Fw	5′-ACCATCTWCACRTRACCCTCTATGAG-3′		[27]
9	RotaA-NSP3Rv	5′-GGTCACATAACGCCCCTATAGC-3′	60	
10	Probe RotaA-NSP3Pr	5′-AGTTAAAAGCTAACACTGTCAAA-3′		

**Table 3:** HAdV and RVA detection with qPCR and cPCR at the four sites.

Site	qPCR HAdV	cPCR HAdV	qPCR RVA
Chelstone	3/5	3/5	3/5
Mass Media	5/5	3/5	4/5
Kaunda Square	5/5	5/5	4/5
Manchichi	5/5	5/5	3/5
Total	18/20 (90%)	16/20 (80%)	14/20 (70%)

**Table 4:** Results for human adenovirus and rotavirus A screening on RT-PCR and conventional PCR, and the sequence details for the human adenovirus hexon gene.

Sample ID	Sampling Site	Week of Collection	RVA qPCR	HAdV qPCR	HAdV cPCR	GenBank Accession no. (HAdV)	% Nucleotide Identity
1	CB	1	Negative	Negative	Negative		
2	MMP	1	POSITIVE	POSITIVE	POSITIVE	PP341455	99.30
3	KSP	1	POSITIVE	POSITIVE	POSITIVE		
4	MP	1	POSITIVE	POSITIVE	POSITIVE	PP341456	98.31
5	CB	2	Negative	Negative	Negative		
6	MMP	2	Negative	POSITIVE	POSITIVE	PP341457	98.92
7	KSP	2	POSITIVE	POSITIVE	POSITIVE	PP341458	98.85
8	MP	2	Negative	POSITIVE	POSITIVE		
9	CB	3	POSITIVE	POSITIVE	POSITIVE	PP341459	99.01
10	MMP	3	POSITIVE	POSITIVE	POSITIVE	PP341460	98.62
11	KSP	3	POSITIVE	POSITIVE	POSITIVE	PP341461	99.53
12	MP	3	POSITIVE	POSITIVE	POSITIVE		
13	CB	4	POSITIVE	POSITIVE	POSITIVE		
14	MMP	4	POSITIVE	POSITIVE	POSITIVE	PP341462	98.48
15	KSP	4	POSITIVE	POSITIVE	POSITIVE	PP341463	99.03
16	MP	4	Negative	POSITIVE	POSITIVE		
17	CB	5	POSITIVE	POSITIVE	POSITIVE		
18	MMP	5	POSITIVE	POSITIVE	POSITIVE	PP341464	99.03
19	KSP	5	Negative	POSITIVE	POSITIVE	PP341465	98.68
20	MS	5	POSITIVE	POSITIVE	POSITIVE		

CB = Chelstone BMFs, MMP = Mass Media PEG, KSP = Kaunda Square PEG, MS = Manchichi skimmed milk.

## Data Availability

The data presented in the study is available on request from the corresponding author.

## Supplementary Materials

The following supporting information can be downloaded at: https://www.mdpi.com/article/10.3390/pathogens13060486/s1, Table S1: Sites showing the results and the methods used for detection of HAdV and RVA.
