# Supplementary material for: Detection of Human Adenovirus and Rotavirus in Wastewater in Lusaka, Zambia: Demonstrating the Utility of Environmental Surveillance for the Community
Source: Pathogens. 2024 Jun 7;13(6):486. doi: 10.3390/pathogens13060486 (PMC11206273; doi:10.3390/pathogens13060486)
Supplement: Supplementary file 1 [file pathogens-13-00486-s001.zip › pathogens-2945979-supplementary.pdf]

Table S1. Sites showing the results and the methods used for detection for detection of HadV, and RVA.

Site	Week	Methods	Adeno qRTPCR	Rota qRTPCR	Site Results HAdV	Site Results RVA
**Chelstone**	- 1	- PEG	-	-	**Negative**	**Negative**
	- 1	- BMFs	-	-		
	- 1	- SM	-	-		
**Mass Media**	- 1	- PEG	+	-	**Positive**	**Positive**
	- 1	- BMFs	-	-		
	- 1	- SM	+	+		
**Kaunda Square**	- 1	- PEG	+	+	**Positive**	**Positive**
	- 1	- BMFs	+	-		
	- 1	- SM	+	+		
**Manchichi**	- 1	- PEG	+	+	**Positive**	**Positive**
	- 1	- BMFs	-	-		
	- 1	- SM	+	+		
**Chelstone**	- 2	- PEG	-	-	**Negative**	**Negative**
	- 2	- BMFs	-	-		
	- 2	- SM	-	-		
**Mass Media**	- 2	- PEG	-	-	**Positive**	**Negative**
	- 2	- BMFs	+	-		
	- 2	- SM	-	-		
**Kaunda Square**	- 2	- PEG	+	-	**Positive**	**Positive**
	- 2	- BMFs	+	+		
	- 2	- SM	+	+		
**Manchichi**	- 2	- PEG	+	-	**Positive**	**Negative**
	- 2	- BMFs	+	-		
	- 2	- SM	+	-		

	Week	Methods	Adeno qRTPCR	Rota qRTPCR	Site Results HAdV	Site Results RVA
**Chelstone**	- 3	- PEG	-	-	**Positive**	**Positive**
	- 3	- BMFs	+	+		
	- 3	- SM	-	-		
**Mass Media**	- 3	- PEG	+	-	**Positive**	**Positive**
	- 3	- BMFs	-	-		
	- 3	- SM	-	+		
**Kaunda Square**	- 3	- PEG	+	-	**Positive**	**Positive**
	- 3	- BMFs	+	-		
	- 3	- SM	+	+		
**Manchichi**	- 3	- PEG	+	+	**Positive**	**Positive**
	- 3	- BMFs	+	+		
	- 3	- SM	+	+		
**Chelstone**	- 4	- PEG	+	+	**Positive**	**Positive**
	- 4	- BMFs	-	-		
	- 4	- SM	+	+		
**Mass Media**	- 4	- PEG	-	-	**Positive**	**Positive**
	- 4	- BMFs	+	+		
	- 4	- SM	-	-		
**Kaunda Square**	- 4	- PEG	+	+	**Positive**	**Positive**
	- 4	- BMFs	+	+		
	- 4	- SM	+	+		
**Manchichi**	- 4	- PEG	+	-	**Positive**	**Negative**
	- 4	- BMFs	+	-		
	- 4	- SM	+	-		

	Week	Methods	Adeno qRTPCR	Rota qRTPCR	Site Results HAdV	Site Results RVA
**Chelstone**	- 5	- PEG	+	+	**Positive**	**Positive**
	- 5	- BMFs	+	-		
	- 5	- SM	+	+		

**Mass Media**	- 5	- PEG	-	-	**Positive**	**Positive**
	- 5	- BMFs	+	+		
	- 5	- SM	+	-		
**Kaunda Square**	- 5	- PEG	+	-	**Positive**	**Negative**
	- 5	- BMFs	+	-		
	- 5	- SM	+	-		
**Manchichi**	- 5	- PEG	+	+	**Positive**	**Positive**
	- 5	- BMFs	+	-		
	- 5	- SM	+	+		
